# Performance of Hybrid Reinforced Composite Substrates in Adhesively Bonded Joints Under Varied Loading Rates

**DOI:** 10.3390/polym17040469

**Published:** 2025-02-11

**Authors:** Hossein Malekinejad, Ricardo J. C. Carbas, Eduardo A. S. Marques, Lucas F. M. da Silva

**Affiliations:** 1 Instituto de Ciência e Inovação em Engenharia Mecânica e Engenharia Industrial (INEGI), Rua Dr. Roberto Frias, 4200-465 Porto, Portugal; hmalekinejad@inegi.up.pt; 2 Departamento de Engenharia Mecânica, Faculdade de Engenharia (FEUP), Universidade Do Porto, Rua Dr. Roberto Frias, 4200-465 Porto, Portugal; emarques@fe.up.pt (E.A.S.M.); lucas@fe.up.pt (L.F.M.d.S.)

**Keywords:** adhesive bonding, composite, delamination, single-lap joint, fiber metal laminate (FML), cohesive zone model (CZM), energy absorption

## Abstract

The use of adhesive bonding for joining composites has grown due to its excellent performance compared to traditional joining methods. However, delamination remains a significant issue in adhesively bonded composite joints, often causing early failure and reducing joint performance. To address this, there is a strong interest in methods that enhance the through-thickness strength of composite substrates to reduce the risk of delamination. Various studies have suggested techniques to prevent delamination in carbon fiber reinforced polymer (CFRP) single-lap joints (SLJs). This study investigates the reinforcement of substrates to prevent delamination, often by adding a tough polymer or metal layer (called fiber metal laminates) to the top and bottom surfaces of the substrates. The effects of incorporating aluminum and film adhesive layers (each comprising 25% of the composite substrate’s thickness) on the failure load and failure mode of bonded joints under different loading rates, including quasi-static (1 mm/min), high-rate (0.1 m/s), and impact (2.5 m/s) conditions, were examined. These configurations were also simulated using cohesive zone modeling (CZM) across all loading rates to predict failure load and mechanisms numerically. Under impact loading, substituting outer CFRP layers with polymer or metal layers significantly increased the failure load and energy absorption capacity. Samples reinforced with aluminum and polymer showed approximately 39% and 13% higher failure loads, respectively, compared to the reference CFRP samples under impact. In terms of energy absorption, SLJs reinforced using aluminum could dissipate energy about 15% greater than the reference CFRP SLJs. The polymer reinforcement configuration can enhance specific strength with a relatively smaller increase in weight compared to FML. This is particularly important in aerospace applications, where minimizing weight while improving performance is crucial.

## 1. Introduction

The increasing use of carbon fiber reinforced plastic (CFRP) materials in different industries, due to their unique properties like high strength-to-weight ratio [1,2], makes it important to study their behavior in different composite structures under various loading conditions. Manufacturing such a structure on a large scale faces challenges in terms of transportation. Therefore, composite structures are usually manufactured on a smaller scale and then joined together to form the desired structural geometry [3]. Among all joining methods, the adhesive bonding joints are well known for their advantages over the classical joining method, e.g., welding, fastening and riveting [4,5]. As a result, structural adhesives are widely used to join composites in various industries, including aerospace applications [6]. Different types of adhesively bonded composite joints have been developed for a wide range of applications, with single-lap joints (SLJs) being the most widely studied and commonly used [4,7,8]. The substrates in SLJs are subjected to peel stresses. When it comes to composite substrates, these peel stresses can cause premature delamination failure due to the weakness of composite substrates in the thickness direction, which is a challenge in using adhesive bonding techniques to bond composite substrates in SLJ configurations [9]. To address this weakness in composite SLJs, various approaches have been proposed in the literature. These approaches can be categorized into two groups: adhesive layer modifications and substrate modifications. To modify the adhesive, well-known techniques include using a mixed adhesive approach and adding nano additive particles [10,11]. The methods that have been used to avoid delamination by reinforcing composite substrates are namely using thin plies [12], changing stacking sequences [13], creating curvature in substrates [14] and applying a corner fillet to decrease the stress concentration [15].

Using different materials with different levels of toughness through-the-thickness like fiber metal laminates (FMLs) is one of these approaches. FMLs are hybrid composite materials in which CFRP plies are reinforced with a thin metal layer [16,17]. The concept of FMLs was first introduced at the Faculty of Aerospace Engineering at the Delft University of Technology in 1978 during research on the behavior of aluminum alloys under fatigue loading. It was later patented in 1984 and employed in real applications by the Alcoa company [18]. This combination can take advantage of the metal’s intrinsic properties, such as high stiffness, strength, impact resistance, and greater toughness compared to CFRP, while also benefiting from CFRP’s lightweight nature and resistance to corrosion and fatigue [19].

Achieving strong and proper adhesion between metal and fiber remains a significant challenge in FMLs [20]. To address this challenge and enhance mechanical interlocking [21], using sanding [22] and sandblasting [23], chemical treatment [24] with acid and alkaline etching [25,26], electromechanical treatment [27], anodizing, lasering, plasma etching [28,29,30] and sol-gels [31,32] have been widely discussed in the literature. To ensure proper adhesion between the CFRP laminate and the thin sheet of metal alloys, common surface preparation techniques, like phosphoric acid anodizing (PAA) or sulfuric acid–sodium dichromate etching, are used to create suitable bonding surfaces. However, these processes involve hazardous materials and generate waste, making them both environmentally and economically undesirable [33]. An alternative technique is applying a sol-gel pre-treatment, which offers an excellent environmentally friendly option to achieve the required high performance [34].

When it comes to adhesive joints, using FMLs as substrates requires optimization of the percentage of the substrate’s thickness allocated to metal, as this greatly impacts the desired enhancement achieved with FMLs. Additionally, the adhesive used to bond the substrates also influences the performance of FML substrates and must be carefully selected. Morgado et al. [35] investigated the effect of adhesives by bonding FML substrates with two different types and concluded that the benefits of using FML substrates are strongly influenced by the mechanical properties of the adhesive used. Peifei Xu et al. [36] investigated the influence of geometry on the damage behavior of FML adhesive joints, emphasizing the use of acoustic emission for damage assessment. Their results highlight how geometric parameters influence damage propagation in hybrid FML joints and establish a framework linking failure modes to acoustic emission parameters. A study by Mottaghian et al. [37] explored FMLs made with basalt-epoxy laminates and magnesium alloys bonded using epoxy resin, focusing on the effects of surface treatments, adhesive thickness and overlap bond length on joint performance. The investigation revealed that, while increasing overlap length and adhesive thickness improves joint strength up to a certain threshold, exceeding this limit leads to diminishing returns and negatively impacts performance. Although FMLs have been extensively studied across various applications, their potential benefits in adhesive joints still require further exploration. Consequently, the authors initiated their research on adhesive joints reinforced with FML substrates by optimizing the placement of the aluminum layer within the thickness [38]. Initial findings revealed that positioning the aluminum layer on the outer surfaces of the substrates yielded the most significant improvements in strength. Building on this, the authors’ subsequent efforts focused on optimizing the thickness of the aluminum layer and its percentage relative to the total thickness of the substrates [35]. The results of these studies showed that using aluminum as reinforcement can increase the strength of SLJs by approximately 27% and 23% under static and impact loading, respectively. The failure mode was delamination for CFRP-only SLJs, whereas it was cohesive for SLJs reinforced with aluminum. Optimizing aluminum-reinforced SLJs requires a more in-depth study, as multiple factors, such as the intended operating loading rate and overlap length, play a crucial role in the performance of hybrid reinforced SLJs. Therefore, this study aims to enhance the overall strength and toughness of composite SLJs through hybridization. By subjecting the latter identified SLJs to three different loading rates, this assessment becomes more comprehensive, offering valuable insights for future applications of composite adhesive joint structures. To achieve this, the composite substrates were manufactured with three configurations, including an aluminum layer (FML), incorporating a tough polymer layer on the top and bottom surfaces of composite substrates and a reference CFRP substrate SLJ. SLJs fabricated with these configurations were subjected to quasi-static (1 m/min), high-rate (0.1 m/s) and impact (2.5 m/s) loading. In addition to fiber metal laminates (FMLs), this study proposed a novel polymer reinforcement configuration that can enhance specific strength with a relatively smaller increase in weight compared to FML. This is particularly important in aerospace applications, where minimizing weight while improving performance is crucial.

Various approaches have been presented in the literature to model composite SLJs and determine their failure load and mechanisms. In the continuum mechanics method, failure load predictions are typically based on the maximum values of stress [39], strain [40] or plastic energy density [41]. However, this approach can become impractical in certain situations due to the inherent singularity at the edge of bonded joints [42], as it assumes that the material and structure are continuous [42]. However, in fracture mechanics approaches, where the singularity at the crack tip is considered, fracture toughness can be used to predict the strength of bonded joints [43]. Another interesting approach is the Extended Finite Element Method (XFEM). According to a study by Campilho et al. [44], it was found that crack initiation in the adhesive layer could not be predicted using this method. However, Xara et al. [45] studied how different XFEM damage initiation criteria affected the strength prediction of hybrid single-lap bonded joints. Their findings indicated that XFEM provided accurate predictions when employing either the quadratic nominal stress or maximum nominal stress criteria. CZM, being a progressive damage modeling technique, can predict both crack initiation and propagation without the need for remeshing or additional calculation costs. In this study, a numerical simulation using cohesive elements has been developed to model the failure load and mechanisms for all tested SLJs under the proposed loading rates. This model successfully predicted the failure load and mechanisms without requiring complex damage models.

## 2. Experimental Study Details

This study involved testing multiple single-lap joints with various substrate configurations under different loading conditions. The joints were subjected to quasi-static (1 mm/min), high-rate (0.1 m/s) and impact (2.5 m/s) test conditions. This section outlines the materials, manufacturing process and testing conditions used.

### 2.1. Materials

#### 2.1.1. Adhesive

An epoxy-based structural adhesive, commercially known as 3 M Scotch-Weld AF 163-2K (3M, Saint Paul, MN, USA), was used in film form to manufacture the single-lap joints (SLJs) for this study. This film adhesive facilitates a clean manufacturing process and allows precise control of the bond line thickness, maintained at 0.2 mm through the use of calibrated shims. The curing cycle followed the manufacturer’s recommendation: 130 °C for 1 h. In a previous study [35], the material properties of the film adhesive used were characterized under various loading rates, including quasi-static and impact at a load speed of 3 m/s. However, the properties under high-rate (0.1 m/s) and impact (2.5 m/s) testing were obtained using linear interpolation, as summarized in Table 1.

#### 2.1.2. Substrates

The unidirectional prepreg carbon–epoxy composite, identified by the commercial product code Texipreg HS 160 T700 (Seal Spa, Legnano, Italy), with a ply thickness of 0.15 mm, was used as the CFRP substrate. The material properties used for simulating the CFRP in the numerical study were obtained from [35,46] and are summarized in Table 2 and Table 3. To achieve greater accuracy in the adhesive properties and numerical simulation, linear interpolation was applied to determine the CFRP properties at varying loading speeds. The orthotropic properties of CFRP were described in terms of the X, Y and Z coordinates, corresponding to the fiber, transverse and thickness directions of the composite substrates, respectively.

The aluminum alloy used for reinforcing the CFRP in FMLs was the 2024-T3 alclad series, available in 0.4 mm thickness (AMI Metals, Charleroi, Belgium). This particular aluminum alloy was chosen due to its widespread use in the aerospace industry and the availability of its properties in the literature. The aluminum’s elastic properties are identified by the Young’s modulus of 72 GPa and the Poisson’s ratio of 0.33 [47]. Additionally, strain–stress curves of aluminum alloy (2024-T3 Alclad) presented in [47] were utilized to obtain the elasto-plastic properties of the aluminum alloy for implementing the elasto-plastic modeling.

### 2.2. Configurations and Preparation of Specimens

**Figure 1:**
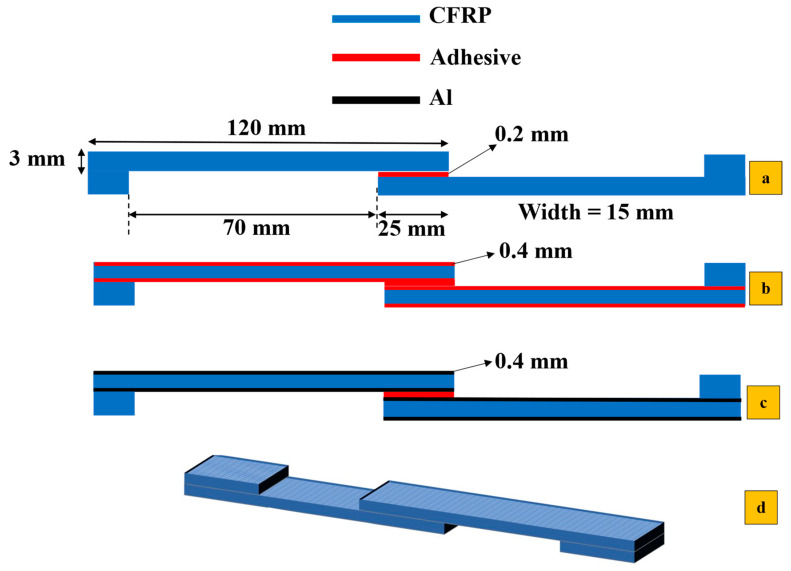
All manufactured configurations (**a**) reference CFRP, (**b**) Adh + CFRP, (**c**) Al + CFRP and (**d**) schematic 3D view of SLJs.

Figure 1a shows the dimensions and various SLJ configurations evaluated in this study. To ensure reliable comparisons, the substrate thickness was kept constant at 3 mm across all configurations, with a 0.2 mm film adhesive layer. As previously mentioned, three different configurations were manufactured and tested in this study (see Figure 1b,c), and their results were compared with the reference CFRP SLJs (see Figure 1a). In configurations labeled b and c, a thin 0.4 mm aluminum layer and two layers of film adhesive (with a total 0.4 mm thickness) were applied to both the top and bottom surfaces of the CFRP substrates, respectively. The reference SLJs are named CFRP. SLJs reinforced with aluminum are labeled Al + CFRP. Those reinforced with two layers of polymer (film adhesive) on each surface are marked as Adh + CFRP.

To manufacture the identified SLJ configurations, CFRP prepreg plies were stacked layer by layer using the hand lay-up method to achieve the desired thickness corresponding to each configuration. The stacked CFRP laminates were then used as substrates in the adhesively bonded joints and bonded together to form the SLJ structure by applying a layer of film adhesive. An aluminum mold was used to control the thickness of substrates and adhesives. The co-curing method was employed to facilitate the manufacturing process.

Since achieving good adhesion between aluminum, CFRP and adhesive can be challenging, an additional step is required to manufacture the SLJs reinforced with aluminum. Proper adhesion between the aluminum and CFRP in Al + CFRP was ensured by applying surface treatment to the aluminum. This treatment starts with cleaning the abrased aluminum surface using acetone to adequately eliminate all contaminants from the bonding surfaces. Cleaning the surface of aluminum with acetone after using sandpaper does not provide enough adhesion. An additional surface treatment, such as applying the sol-gel 3 M Surface Pre-Treatment AC-130–2, needs to be applied. Employing the sol-gel chemical surface treatment can decrease adhesive failure and improve load-bearing capacity by increasing the roughness of the surface chemically. Consequently, in this study, sol-gel AC-130–2 was manually applied generously with a brush and allowed to dry for at least one hour, following the supplier’s instructions [48].

### 2.3. Testing Condition

**Figure 2:**
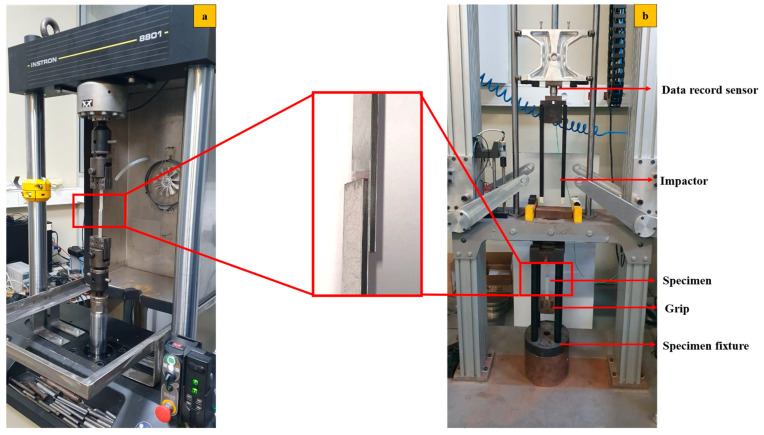
Test set up (**a**) hydraulic testing machine and (**b**) custom-built drop-weight testing machine.

All aforementioned configurations were tested under displacement control, with crush head speeds set to quasi-static (1 mm/min), high-rate 0.1 m/s and impact (2.5 m/s). A servo-hydraulic machine (Instron 8801 sourced from Norwood, MA, USA) with a 100 kN load cell capacity was used for the quasi-static and high-rate tests, while the impact test was conducted using a custom-built drop-weight testing machine (see Figure 2). All tests were carried out under ambient conditions (room temperature of 24 °C, relative humidity of 55%). For static and high-rate loading speeds, at least three samples were tested, ensuring acceptable discrepancies due to the small standard deviation of the results. For impact loading, at least five samples were tested for each configuration due to the greater variability observed.

## 3. Numerical Study Details

Finite element analysis using the Abaqus software 2017, was conducted to better understand the performance of identified configurations under different loading rates. In this section, the details of the numerical study, as well as the failure criteria, are presented.

**Figure 3:**
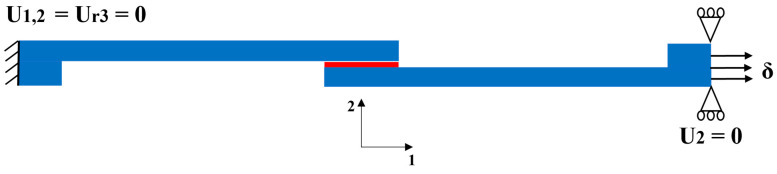
Applied load and boundary conditions for the simulation of identified SLJs.

To simplify and reduce the calculation cost, 2D models with quasi-static, high-rate, and impact loading were used. A static general analysis was employed for quasi-static loading, simulating a near-zero load speed rate by applying a slow, quasi-static displacement. For high-rate and impact scenarios, dynamic explicit analyses were used, where the rate was simulated by varying the velocity of the applied displacement, corresponding to faster loading rates. For reference CFRP specimens, the composite substrates were modeled using the engineering constants. The aluminum, used as reinforcement in Al + CFRP, was modeled as an elasto-plastic material, while for Adh + CFRP, the polymer reinforcement layer was modeled using CZM along with the elastic behavior. All assigned rate-dependent material properties are presented in Table 1, Table 2 and Table 3. Figure 3 illustrates the applied displacement and boundary conditions for the simulated SLJs. The left end was fixed in all directions, while the right end was constrained in the thickness direction of the SLJs, with the load applied at this end in line with the fiber direction of the unidirectional composite substrates. These numerical models allowed for an evaluation of strain-rate-dependent performance and failure in quasi-static, high-rate and impact loading.

**Figure 4:**
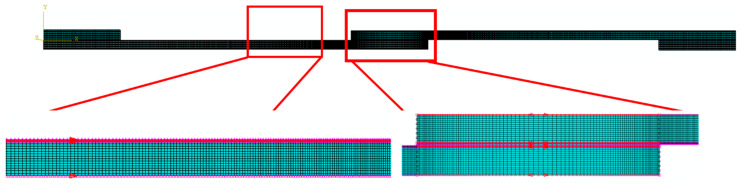
Assigned mesh for SLJs.

Regarding the mesh shown in Figure 4, quadrilateral plane strain elements with reduced integration were assigned. Cohesive elements of the COH2D4 type were assigned to the location where cohesive elements were applied.

**Figure 5:**
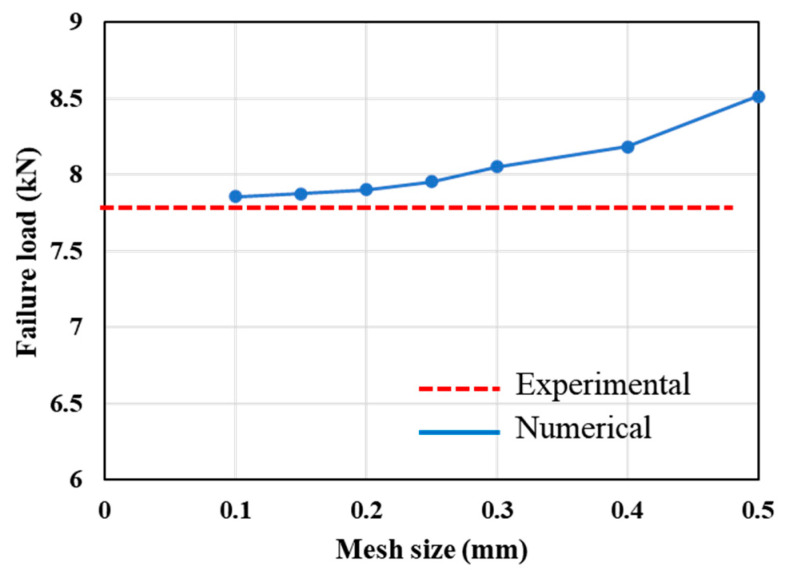
Influence of mesh size on the failure load predicted numerically.

Moreover, to assess the effect of mesh size on the numerically predicted failure load, a mesh convergence study was conducted on the reference CFRP-only SLJ under quasi-static loading conditions. The thickness of cohesive layers (direction 2 as shown in Figure 3) located within the CFRP substrates and adhesive bond line were restricted to only one raw cohesive element, ensuring the convergence of the model. However, in direction 1 based on Figure 3, the mesh convergency study was performed. For this analysis, both single- and double-biased mesh distributions were employed along the bond line and the substrates, respectively (see Figure 4). In the biased mesh method, the maximum and minimum mesh sizes are defined at both ends of the selected edge based on the importance of each side in terms of load transfer and stress concentration, with the distribution of elements between these two ends being linear. The maximum mesh size, set at regions of lower importance, was consistently maintained at 0.5 mm. Minimum mesh sizes of 0.1 mm, 0.15 mm, 0.2 mm, 0.25 mm, 0.3 mm, 0.4 mm and 0.5 mm were evaluated to study their influence on the numerical results. The detailed effects of these mesh sizes are illustrated in Figure 5. Based on the results presented in Figure 5, a minimum mesh size of 0.1 mm was determined to provide an effective balance between the accuracy of finite element simulations and computational efficiency, allowing reliable comparisons with experimental data. Consequently, the selected mesh sizes for the numerical models were 0.1 mm for the bondline and 0.5 mm for the substrates. These mesh sizes were consistently applied across all numerical simulations to ensure uniformity and comparability.

### 3.1. Elastic Model Analysis

The numerical study was initially started by simulating all the identified configurations without applying failure criteria, focusing solely on stress analysis and evaluating their behavior under quasi-static loading conditions. The maximum principal stress distribution for each configuration was analyzed and compared to the reference CFRP sample to determine whether adding a tough aluminum layer and adhesive caused a reduced stress concentration. This analysis was performed under load control. For the reference CFRP model, a load of 7.6 kN was applied, while for the Al + CFRP and Adh + CFRP models, an 8 kN load was used, corresponding to the experimentally determined failure loads.

### 3.2. CZM Analysis

A cohesive zone model (CZM) was applied, utilizing a triangular traction–separation law. This model, with parameters defined in Table 1 and Table 3, was used to estimate the failure load and mechanism and also compare load–displacement curves from both experimental and numerical perspectives.

**Figure 6:**
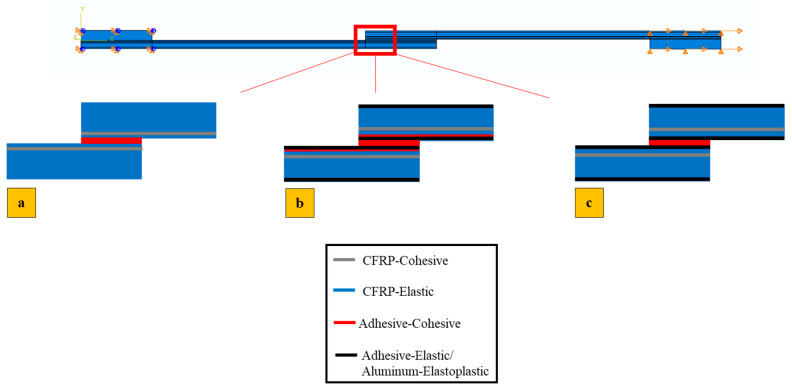
Schematic of introducing cohesive in (**a**) CFRP reference, (**b**) Adh + CFRP and (**c**) Al + CFRP.

As illustrated schematically in Figure 6, a thin cohesive element layer with a thickness of 0.075 mm (equal to half the thickness of each CFRP prepreg layer) was incorporated within the CFRP to predict delamination for the reference CFRP specimens. For the Al + CFRP and Adh + CFRP specimens, this cohesive layer was increased to 0.15 mm, matching the full thickness of one CFRP prepreg layer. Additionally, the bond line adhesive was modeled using a cohesive zone model (CZM).

In the configuration of Adh + CFRP, the delamination might be growth through the reinforced layer with adhesive; consequently, to simulate delamination within the reinforcement polymer layer, a thin cohesive layer with a thickness of 0.075 mm was also added. In addition, the adhesive that bonds the substrates was also simulated using CZM. For the reference CFRP samples across all simulated loading rates, the cohesive layer for predicting delamination was placed as close as possible to the adhesive–substrate interface, at a distance of 0.1 mm. This positioning was selected to align with experimental observations of delamination initiation, with the spacing constrained by numerical convergence requirements. In contrast, for the Al + CFRP and Adh + CFRP specimens, the cohesive layer was positioned 0.4 mm above the interface between the adhesive layer and the substrates. To assign the identified material properties of aluminum, adhesive and cohesive layers, the respective sections corresponding to each material, as shown in Figure 6, were partitioned using face partitions.

## 4. Results and Discussions

This section presents numerical and experimental findings across a range of loading rates, from quasi-static to impact. It also compares the numerically and experimentally observed failure surfaces and loads. Additionally, it discusses the results from the finite element analysis conducted to determine the stress distributions under quasi-static loading conditions. Finally, it evaluates the performance of the identified SLJs in terms of average strength and energy absorption.

### 4.1. Numerical Stress Analysis

**Figure 7:**
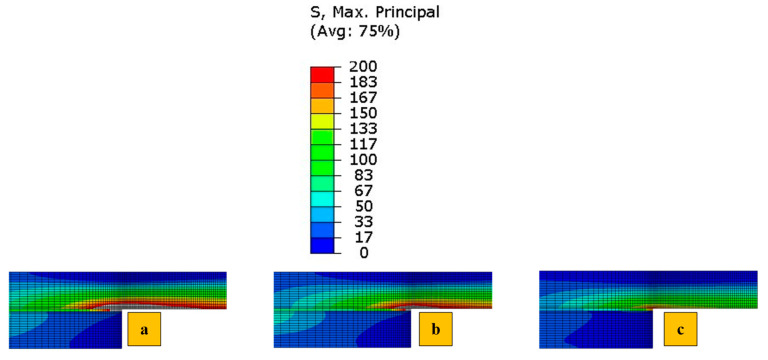
Stress analysis under static loading for (**a**) reference CFRP SLJs, (**b**) Adh + CFRP SLJs and (**c**) Al + CFRP SLJs (elements with stress levels exceeding 200 MPa are displayed in gray).

This study began with the development of an elastic finite element model for all identified configurations. The primary objective of this numerical analysis was to evaluate the influence of reinforcing CFRP substrates with aluminum and polymer layers on the stress distribution. In this part of the numerical study, all configurations were analyzed under quasi-static loading, and the maximum principal stresses are shown in Figure 7, which examines the effect of reinforcement on the stress field. The detailed numerical study, in which the cohesive zone model was employed to predict the behavior of the tested specimens, was discussed in this manuscript in detail. Due to the symmetry of the joints and stress distribution, only half of the overlap region is shown. For easier visual comparison, the stress range is limited to between 0 and 200 MPa across different load levels in Figure 7. Elements with stress levels exceeding 200 MPa are displayed in gray. As expected, the inherent ductility of polymer and aluminum, applied as reinforcement, likely reduces stress concentrations, allowing them to deform plastically and consequently achieve a more effective load transfer than a stiffer and brittle material like CFRP. The Adh + CFRP configuration likely shows higher stress concentrations than Al + CFRP because the film epoxy adhesive used, after curing, is not as ductile as aluminum; while effective for bonding, it may not distribute stress as uniformly. In general, the reference CFRP model tends to show higher stress concentrations due to its lower ductility compared to aluminum and polymer. This conclusion makes it interesting to evaluate the proposed configuration experimentally and under higher loading rates, as the rate dependency of aluminum and polymer could make the enhancement in performance more promising.

### 4.2. Comparison of Experimental and Numerical Results

In this subsection, the failure loads and mechanisms obtained experimentally and numerically are compared to evaluate the effectiveness of the proposed cohesive zone model.

**Figure 8:**
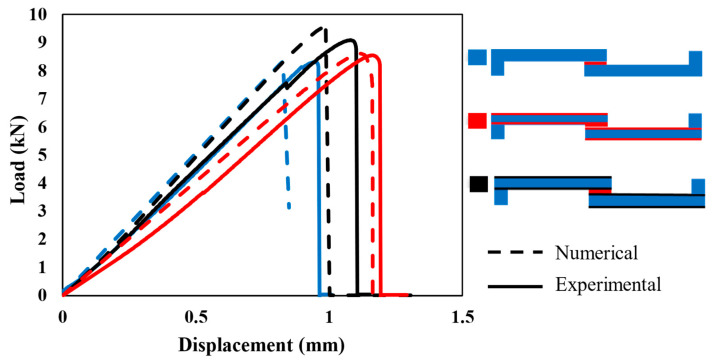
Comparison of numerical (dashed) and experimental (solid) load–displacement curves under the quasi-static (1 mm/min) loading condition for all configurations.

Figure 8 illustrates the behavior of each configuration under quasi-static loading (1 mm/min). The experimental (solid line) and numerical (dashed line) curves show a good correlation in terms of initial stiffness and overall load–displacement behavior across all configurations.

The stiffness of each configuration, determined from the slope of the elastic region in the curves, shows that Adh + CFRP samples are less stiff. This is expected, as replacing the CFRP layer with polymer, which has a much lower modulus, makes the SLJs more deformable and reduces the stiffness. The stiffness of Al + CFRP samples and CFRP-only samples is comparable, which confirms the effectiveness of the FML concept without reducing the strength of the SLJs. Moreover, both AL + CFRP and Adh + CFRP SLJs exhibit slightly higher failure loads and displacements at failure compared to reference CFRP-only SLJs.

**Figure 9:**
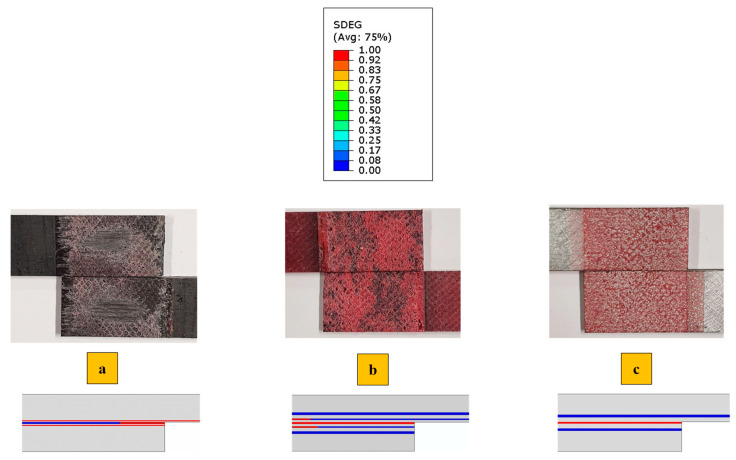
Failure mechanism obtained experimentally and numerically under the quasi-static (1 mm/min) loading condition for (**a**) CFRP, (**b**) Adh + CFRP and (**c**) Al + CFRP.

Figure 9 illustrates the comparison between the failure mechanisms observed experimentally and those obtained numerically at the failure load for all configurations. It was found that, similar to the failure load, the failure mechanisms were accurately predicted using the proposed model. This correlation is evident between the “SDEG” failure parameter of the cohesive elements (shown in Figure 9), highlighted in red to indicate elements where failure occurs, and the experimentally observed failure surfaces.

**Figure 10:**
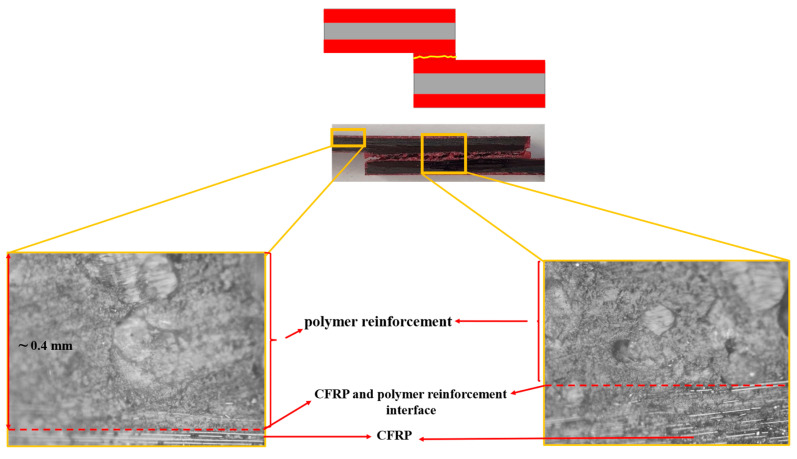
Crack path based on the microscopic image for Adh + CFRP configuration under quasi-static loading.

Under quasi-static loading, cohesive failure was observed across all configurations. However, for the CFRP-only reference, a small area experienced delamination within the CFRP layers, very close to the interface. In Adh + CFRP SLJs, a small portion of delamination also occurred within the polymers reinforced layers. The microscopic image shown in Figure 10 includes a lateral view of the Adh + CFRP samples after quasi-static tests, with the crack path schematically highlighted by a yellow line. The crack initiates at the adhesive bond line and remains a cohesive failure along the entire bond line until the final failure.

**Figure 11:**
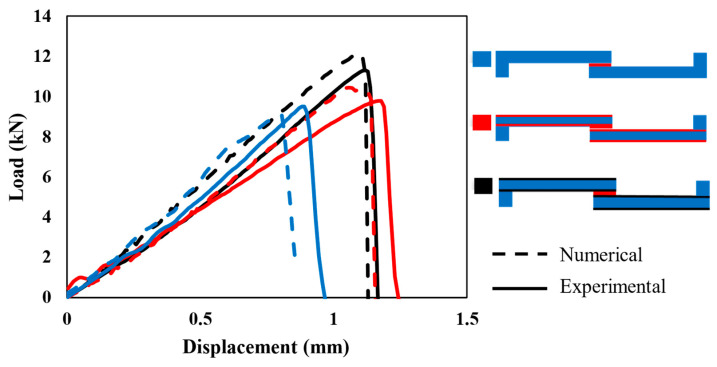
Comparison of numerical (dashed) and experimental (solid) load–displacement curves under the high-rate (0.1 m/s) loading condition for all configurations.

As the loading rate increases to 0.1 m/s, the differences between the numerical and experimental curves become more noticeable, as shown in Figure 11. The high-rate loading introduces strain-rate effects, which the numerical models may not fully capture, especially if rate-dependent properties are not accurately implemented. In this study, the rate dependence of material properties was determined through extrapolation and interpolation, which could be a potential source of error. Regarding the change in the stiffness of reinforced SLJs compared to CFRP-only SLJs, the trend obtained is similar to the quasi-static loading mentioned before: less stiffness for Adh + CFRP SLJs and comparable stiffness for the two other identified SLJs.

**Figure 12:**
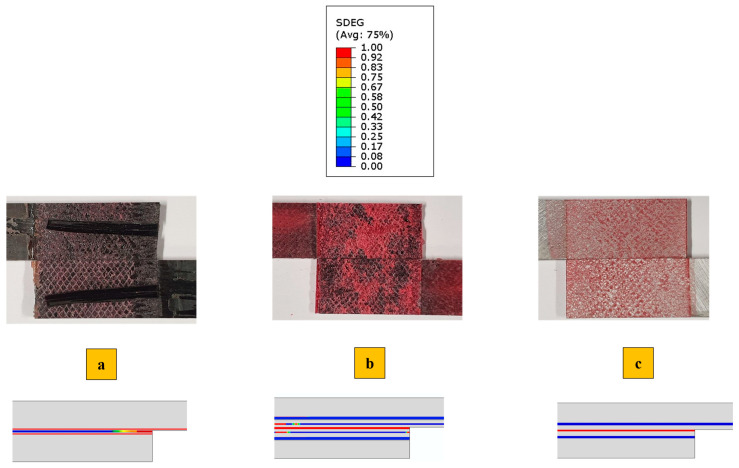
Failure mechanism obtained experimentally and numerically under the high-rate (0.1 m/s) loading condition for (**a**) CFRP, (**b**) Adh + CFRP and (**c**) Al + CFRP.

**Figure 13:**
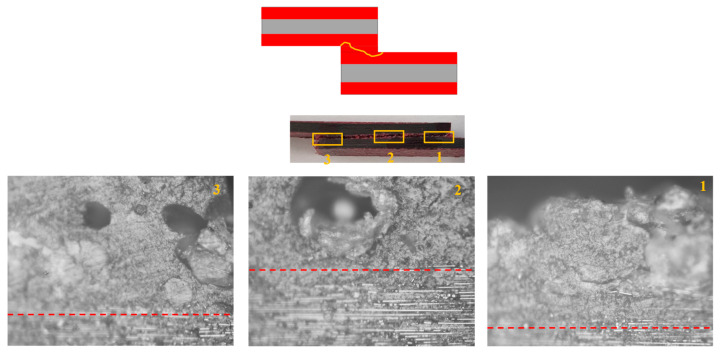
Crack path based on the microscopic image for Adh + CFRP configuration under high-rate loading, CFRP and polymer reinforcement interface shown with red dashed line. (Microscopic images taken from the bottom substrate).

The failure mechanism obtained from high-rate loading rate numerically and experimentally is depicted in Figure 12. Under high-rate loading, the failure of CFRP-only SLJs exhibited severe delamination, resulting in lower strength compared to AL + CFRP SLJs, which failed with a cohesive failure mechanism. The lateral view of the Adh + CFRP samples under high-rate loading is shown in Figure 13, taken using an optical microscope. The crack initiates within the reinforcement polymer layer and, after propagating through a straight portion, moves upward through the adhesive bond line. Eventually, it progresses to final failure within the polymer reinforcement region of the top substrate.

**Figure 14:**
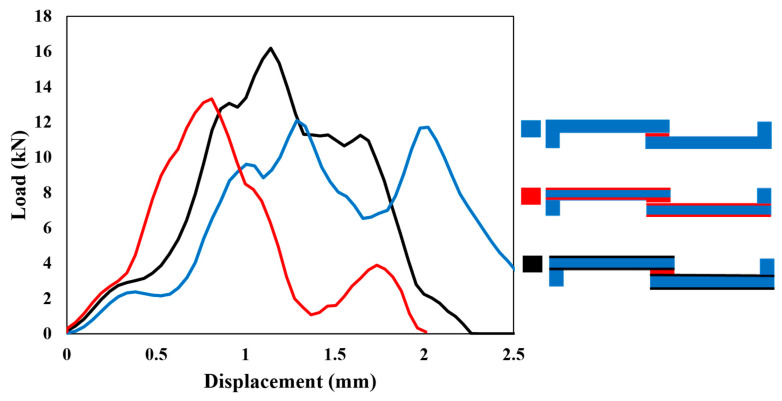
Representative load–displacement curves obtained experimentally under the impact (2.5 m/s) loading condition for all configurations.

**Figure 15:**
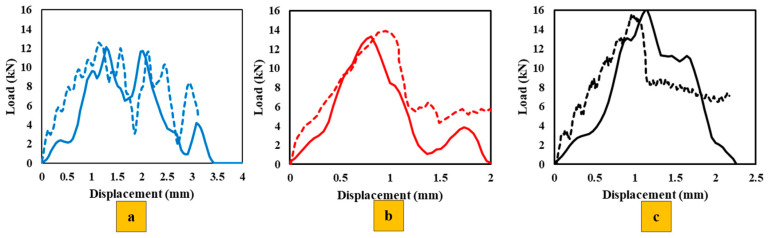
Comparison of numerical (dashed) and experimental (solid) load–displacement curves under the impact (2.5 m/s) loading condition for (**a**) CFRP, (**b**) Adh + CFRP and (**c**) Al + CFRP.

To better understand the complex behavior of the load–displacement curves under impact, the experimental representative load–displacement curves are initially presented in Figure 14. Following this, the numerical results are compared with each curve individually in Figure 15.

Under impact, the complexity of the rate-dependent material behavior likely explains the less predictable load–displacement behavior. Consequently, the discrepancies between experimental and numerical results become more significant (see Figure 15). Experimental curves often show a rapid load rise with a distinct peak, followed by a complex post-peak behavior, which the numerical model may struggle to replicate. Impact loading introduces significant strain-rate sensitivity and inertia effects, which are challenging to replicate accurately in numerical models. However, substituting the traditional linear or bilinear cohesive laws with more advanced models, such as exponential or polynomial laws, or deriving an accurate cohesive law that more effectively captures the nonlinear behavior under dynamic loading, can improve the accuracy and reliability of numerical results.

**Figure 16:**
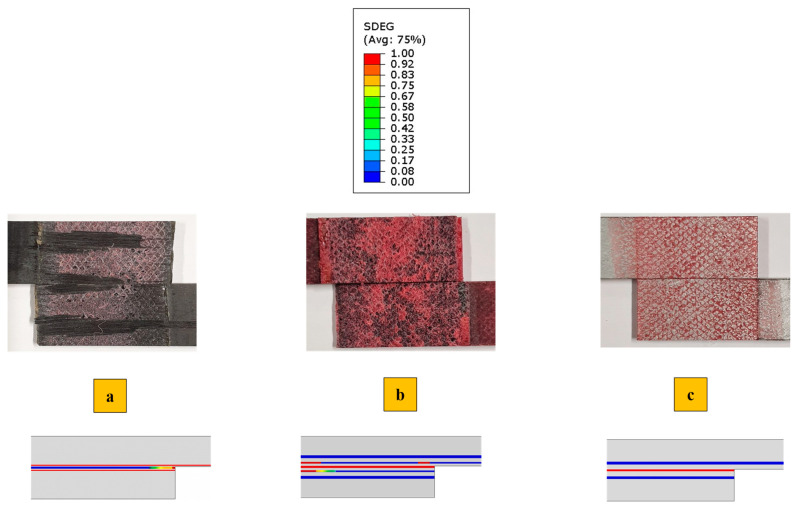
Failure mechanism obtained experimentally and numerically under the impact (2.5 m/s) loading condition for (**a**) CFRP, (**b**) Adh + CFRP and (**c**) Al + CFRP.

**Figure 17:**
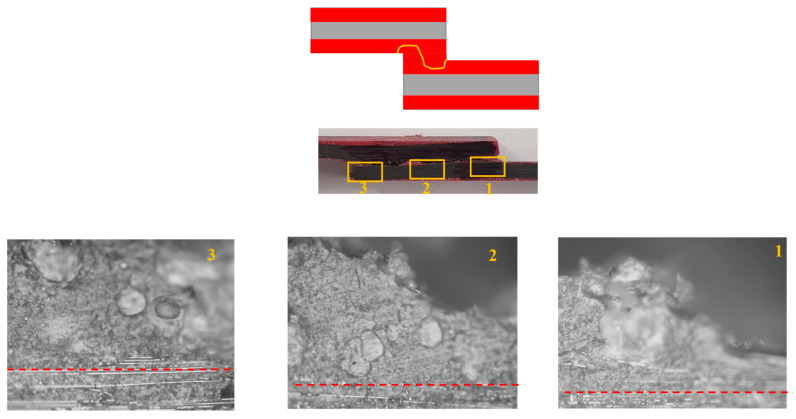
Crack path based on the microscopic image for Adh + CFRP configuration under impact loading, CFRP and polymer reinforcement interface shown with red dashed line. (Microscopic images taken from the bottom substrate).

In Figure 16, the failure mechanisms obtained experimentally and numerically for impact loading are shown and compared. Delamination increased and became more severe in CFRP-only SLJs as the loading rate increased. In contrast, failure in AL + CFRP SLJs remained cohesive, while the Adh + CFRP samples, as indicated by the SDEG index, experienced greater delamination compared to other tested loading rates. As illustrated in Figure 17, the crack initiates close to the CFRP of the bottom substrate within the polymer reinforcement layer. It then propagates upward through the adhesive bond line and ultimately grows close to the CFRP of the top substrate within the polymer reinforcement layer of the top substrate.

In general, a closer examination of the failure surfaces reveals that, in CFRP samples, regardless of the loading rate, the failure is a combination of cohesive and delamination, while delamination consistently occurs near the interface between the adhesive bond line and composite substrates. However, in the case of quasi-static conditions, cohesive failure is the most predominant failure. As the loading rate increases, delamination occurs at a greater distance from the bond-line. In other words, for CFRP samples, higher loading rates result in more severe delamination and extensive crack branching, as illustrated in Figure 16a.

The failure of AL + CFRP is cohesive across all loading rates. This cohesive failure in Al + CFRP samples indicates that the adhesive bond effectively withstands the applied loads. Despite the complete prevention of delamination in Al + CFRP SLJs, the Adh + CFRP SLJs only shift the delamination from the CFRP layer to within the polymer reinforcement.

The proposed numerical CZM performs well in predicting failure loads and modes across all loading rates, especially in quasi-static and intermediate loading, with only minor discrepancies due to model simplification or material property variations. However, under impact loading, cohesive element behavior might diverge, leading to discrepancies in predicting exact post failure load behavior of the load–displacement curves.

### 4.3. Performance Comparison of SLJ Configurations

**Figure 18:**
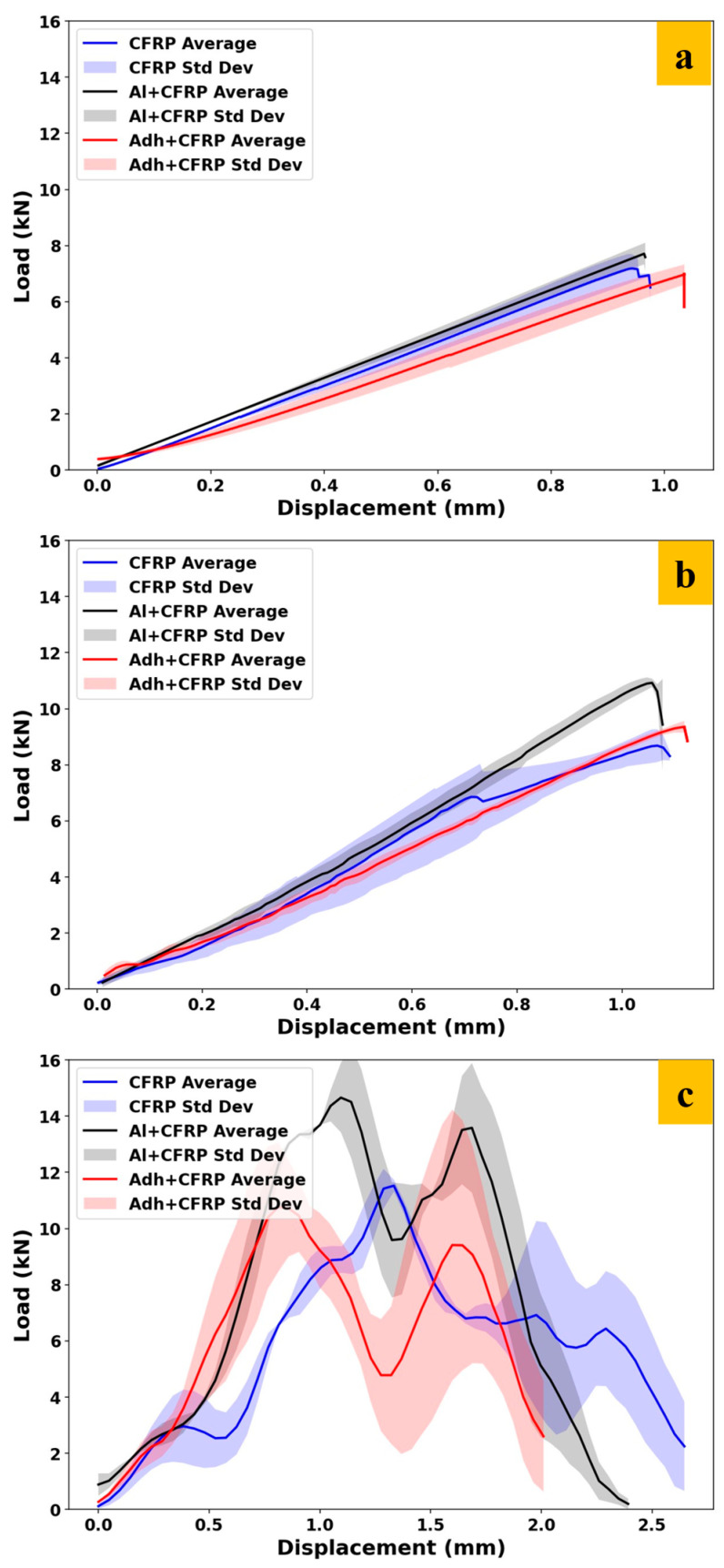
Average load–displacement curves with standard deviation for (**a**) quasi-static (**b**) high-rate and (**c**) impact.

Average load–displacement curves with standard deviations for the three configurations (CFRP, Adh + CFRP, and Al + CFRP), were evaluated under quasi-static, high-rate, and impact loading conditions in Figure 18.

Across all loading rates, the Al + CFRP configuration consistently exhibits superior performance in terms of load-carrying capacity and energy absorption, underscoring the critical role of the aluminum layer. The Adh + CFRP configuration highlights the beneficial impact of adhesive layers in enhancing structural integrity, providing a balance between performance and weight.

Furthermore, the strain-rate sensitivity observed in all configurations demonstrates the material’s ability to respond to varying loading conditions, with higher peak loads achieved at increasing strain rates. This behavior emphasizes the importance of selecting configurations based on the anticipated loading conditions in real-world applications [49]. Finally, the increased variability in impact loading highlights the complex and progressive nature of damage mechanisms at higher loading rates.

**Figure 19:**
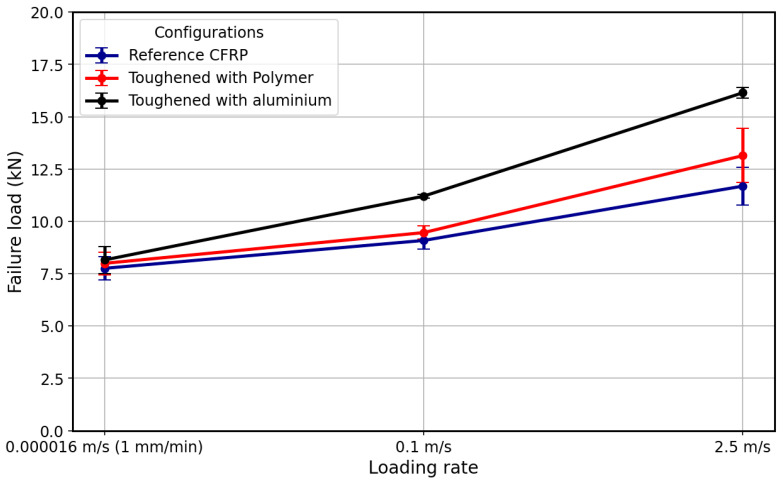
Summary of maximum failure load obtained for each configuration at different loading rates.

The average failure loads for the CFRP-only reference, Al + CFRP and Adh + CFRP SLJs are illustrated in Figure 19. The error bars, which represent the standard deviation for each loading rate, are fairly uniform in size across all data points. This suggests that the testing conditions were well-controlled, leading to consistent results in failure load across samples at each loading rate.

As observed, the load-bearing capacity increases with higher loading rates. This rising trend in failure load can be attributed to the materials strain rate sensitivity, which typically enhances material properties under higher loading rates. At higher loading rates, the material does not allow sufficient time for crack propagation, resulting in an apparent increase in strength. This finding aligns with previous studies, which have shown similar strain rate dependencies, particularly under impact and high-rate loading conditions [50].

The failure load under quasi-static loading is nearly the same across all configurations, with only a slight increase of about 5% for Al + CFRP compared to the CFRP-only reference. In our previous study [38], we analyzed data for CFRP-only and CFRP reinforced with aluminum SLJs with 12.5 mm overlaps under quasi-static loading. The failure mechanism observed in CFRP-only SLJs was cohesive failure, which was similar to the failure mechanism in Al + CFRP samples, resulting in comparable failure loads. Similarly, in this study, the failure mechanism of CFRP samples under quasi-static loading is also mostly cohesive, with delamination happening very close to the interface (see Figure 9a). This can explain the comparable failure loads across all configurations under quasi-static loading. However, the failure mechanism of CFRP-only SLJs may shift to delamination as the overlap length increases to 50 mm. In this case, AL + CFRP SLJs, which exhibit cohesive failure, could demonstrate higher strength compared to the reference samples.

As the loading rate increases, the Al + CFRP and Adh + CFRP configurations exhibit a more significant improvement in strength compare to reference CFRP samples. Under high-rate loading, the Al + CFRP and Adh + CFRP configurations result in approximately 23% and 5% percent improvements, respectively. Additionally, under impact loading, the difference becomes more pronounced, with approximately 39 percent and 13 percent increases for the Al + CFRP and Adh + CFRP samples, respectively.

Furthermore, the Al + CFRP configuration exhibited cohesive failure across all loading rates (see Figure 8, Figure 11 and Figure 15c), effectively reducing delamination. This can be considered as a contributing factor to the higher failure load observed compared to other configurations in which delamination observed. For Adh + CFRP SLJs, in addition to the discussed numerical stress analysis, any defects or inconsistencies that may arise during the manufacturing process in the polymer layer can reduce its load-bearing capacity compared to isotropic aluminum.

Generally, adhesive, aluminum and CFRP materials may exhibit increased stiffness and strength under higher loading rates; consequently, the load level is higher compared to the quasi-static loading condition. Moreover, across all loading rates, the Adh + CFRP consistently performs better than the reference CFRP, achieving higher failure loads. Possible reasons for this increased strength could include higher toughness and more effective load transfer that reinforcing with polymer and aluminum layers causes. The Al + CFRP clearly shows a higher failure load across all loading rates compared to the other configurations. This highlights the effectiveness of designs that use aluminum reinforcement. The Al + CFRP’s superior load-bearing capacity is likely due to the combined benefits of its metal and CFRP components, where the metal layers provide enhanced toughness and energy absorption as shown in Figure 19, while the CFRP layers offer high stiffness and strength.

**Figure 20:**
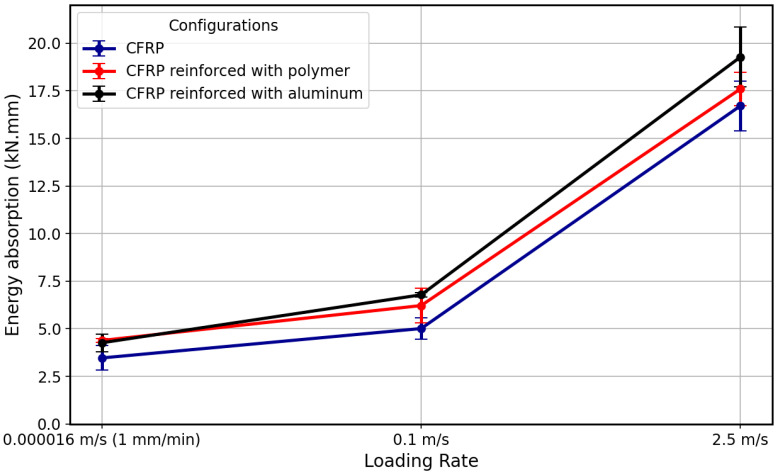
Summary of obtained energy absorption corresponded to each configuration.

Based on the energy absorption capacity of tested configuration shown in Figure 20, the Al + CFRP samples demonstrated the highest energy absorption compared to the other configurations. This enhancement in energy absorption is most pronounced under higher loading rates, where the aluminum reinforcement shows a clear advantage.

The Adh + CFRP samples have higher energy absorption compared to the reference CFRP SLJs, which can be attributed to the additional toughening effect provided by the polymer layer, which improves the overall load transfer and damage tolerance of the composite. The improvement in energy absorption of Adh + CFRP, although notable, is less than that of the aluminum-reinforced composite, likely due to the more brittle nature of the cured epoxy at higher loading rates. The enhancement in energy absorption with increasing loading rates was most pronounced in the aluminum-reinforced samples. As the loading rate increased, the ability of aluminum to absorb energy improved significantly, further emphasizing the material’s rate-dependent behavior.

## 5. Conclusions

This study presents a thorough investigation of composite single-lap joints (SLJs) reinforced with polymer and aluminum under varying loading rates. As anticipated, increasing the loading rate improved the material properties and raised the failure load. All the configurations that were manufactured and tested were also modeled numerically, and the failure load and mechanisms obtained from the numerical simulations were compared with the experimental results. Based on this investigation, the following key conclusions have been drawn.

- In the SLJs reinforced with aluminum, the metal layers can help slow down or prevent crack propagation that typically occurs in brittle composite layers under high-stress conditions. This combination of metal and composite materials allows the Al + CFRP to better withstand impact and high loading rates, as the ductile metal component absorbs energy, thereby reducing stress concentration in the composite layers. This combination not only increases the overall strength of the material but also makes it more resilient to damage under varying loading conditions, which is particularly valuable in applications requiring high-impact resistance, like aerospace or automotive structures.
- The composite SLJs reinforced with aluminum experience approximately 39% and 23% higher strength compared to reference CFRP under impact and high-rate loading, respectively.
- This enhancement was recorded at 13% and 5% for the composite SLJs reinforced with polymers.
- The energy absorption of the composite SLJs reinforced with aluminum is higher, especially as the loading rate increases, compared with the other configurations.
- While the aluminum-reinforced SLJs completely prevent delamination, the polymer-reinforced samples only shift the delamination from the CFRP within the polymer reinforcement.
- Both reinforcement methods reduce stress concentration at the overlap corners and result in a more uniform stress distribution and load transfer compared to the CFRP-only SLJs. Therefore, these two configurations have higher failure load and less delamination.
- The CZM model performs well for all configurations under both quasi-static and high-rate loading. However, as the loading rate increases, the model’s accuracy decreases due to the need for more precise rate-dependent material properties.

## Figures and Tables

**Table 1:** Mechanical properties of film AF 163-2K [35].

Properties	Quasi-Static (1 mm/min)	High-Rate (0.1 m/s)	Impact (2.5 m/s)
Maximum tensile strength (MPa) *	46.93	46.93	46.93
Young’s modulus (MPa) *	1520	1520	1520
Maximum shear strength (MPa)	46.86	46.68	42.3
Shear modulus (MPa) *	565	565	565
Mode I fracture toughness (N/mm)	4.05	4.12	5.72
Mode II fracture toughness (N/mm)	9.77	10.07	17.24

* Assumed to remain constant.

**Table 2:** CFRP elastic properties [46].

*E_x_* (MPa)	*E_y_* (MPa)	*E_z_* (MPa)	*ν_xy_*	*ν_yz_*	*ν_xz_*	*G_xy_* (MPa)	*G_yz_* (MPa)	*G_zx_* (MPa)
109,000	8819	8819	0.342	0.342	0.38	4315	4315	3200

**Table 3:** Cohesive parameters of CFRP [35].

Properties	Quasi-Static	High-Rate	Impact
Maximum tensile strength (MPa) *	40	40	40
Maximum shear strength (MPa) *	35	35	35
Mode I fracture toughness (N/mm)	0.59	0.58	0.42
Mode II fracture toughness (N/mm)	1.2	1.19	0.88

* Assumed to remain constant.

## Data Availability

The original contributions presented in this study are included in the article.
